# Carbapenem-Resistant and Extensively Drug-Resistant Gram-Negative Healthcare-Associated Infections in a Romanian Tertiary Infectious Diseases Hospital: Phenotypic Landscape, Stewardship Gaps and Outcomes (2024–2025)

**DOI:** 10.3390/antibiotics15101003

**Published:** 2026-10-10

**Authors:** Răzvan Roșca, Călin Muntean, Mirela Frandeș, Ruxandra Laza, Daian-Ionel Popa, Victor Bogdan Buciu, Cătălin Prodan-Bărbulescu, Mădălina-Ianca Șuba, Sorina Maria Denisa Laitin

**Affiliations:** 1 Doctoral School, “Victor Babeș” University of Medicine and Pharmacy, E. Murgu Square, No. 2, 300041 Timișoara, Romania; razvan.rosca@umft.ro (R.R.); victor.buciu@umft.ro (V.B.B.); 2 Department for the Prevention and Control of Healthcare-Associated Infections (CPIAAM), Clinical Hospital of Infectious Diseases and Pulmonology “Dr. Victor Babeș” Timișoara, Gheorghe Adam Street No. 13, 300310 Timișoara, Romania; 3 Department III Functional Sciences, Discipline of Medical Informatics and Biostatistics, “Victor Babeș” University of Medicine and Pharmacy, 300041 Timișoara, Romania; 4 Department XIII, Discipline of Infectious Diseases, “Victor Babeș” University of Medicine and Pharmacy Timișoara, E. Murgu Square, Nr. 2, 300041 Timișoara, Romania; laza.ruxandra@umft.ro (R.L.); madalina.suba@umft.ro (M.-I.Ș.); 5 Research Center for Medical Communication, “Victor Babeș” University of Medicine and Pharmacy, 300041 Timișoara, Romania; daian-ionel.popa@umft.ro; 6 Department I, Discipline of Anatomy and Embryology, Faculty of Medicine, “Victor Babeș” University of Medicine and Pharmacy Timișoara, Eftimie Murgu Square 2, 300041 Timișoara, Romania; catalin.prodan-barbulescu@umft.ro; 7 Methodological and Infectious Diseases Research Center, Faculty of Medicine, “Victor Babeș” University of Medicine and Pharmacy, 300041 Timișoara, Romania; 8 Department of Epidemiology, “Victor Babeș” University of Medicine and Pharmacy, Eftimie Murgu Square 2, 300041 Timișoara, Romania; laitin.sorina@umft.ro

**Keywords:** healthcare-associated infections, carbapenem resistance, carbapenem-resistant Enterobacterales, extensively drug-resistant, *Klebsiella pneumoniae*, *Acinetobacter baumannii*, antimicrobial stewardship, antimicrobial resistance surveillance, Romania

## Abstract

Background/Objectives: Romania reports among the highest rates of carbapenem-resistant Gram-negative bacteria in the European Union, but patient-level data on healthcare-associated infections (HAIs) are scarce. We characterised carbapenem resistance, in-hospital outcomes, and carbapenem prescribing in a 24-month HAI cohort. Methods: This was a retrospective study of 315 patients with microbiologically confirmed HAI (2024–2025) at a tertiary infectious diseases hospital, contributing 243 Gram-negative isolates. Carbapenem resistance was determined by the hospital laboratory; extensively drug-resistant (XDR) status, defined as susceptibility to no more than two of the antimicrobial classes tested, was assigned under the hospital surveillance protocol. Risk-factor analysis was exploratory. Results: Of 243 Gram-negative isolates, 162 (66.7%) were carbapenem-resistant and 150 of these (92.6%) were designated XDR. Resistance reached 90.6% in *Klebsiella pneumoniae* and 83.0% in *Acinetobacter baumannii*, against 33.3% in *Pseudomonas aeruginosa* and 0% in *Escherichia coli*, and occurred at every infection site. No routinely recorded characteristic distinguished resistant from susceptible infection after adjustment (area under the curve 0.62). In-hospital death was associated with intensive care transfer (adjusted odds ratio 6.92, 95% confidence interval 3.33–14.38) but not with carbapenem resistance (1.14, 0.52–2.50). Of 92 carbapenem recipients, 54 (58.7%) had a carbapenem-resistant isolate, against which carbapenems are inactive, and 12 (13.0%) had a Gram-negative isolate that was neither carbapenem-resistant nor extended-spectrum β-lactamase-producing. Conclusions: At this centre, carbapenem resistance in Gram-negative HAI is endemic, could not be predicted from the routinely recorded characteristics examined, and exceeds European surveillance figures only among the Enterobacterales. No independent association with mortality was detected, although the susceptible comparison group was small. The XDR proportion was assigned under the hospital surveillance protocol and is not directly comparable with category-based estimates. Because antibiotic exposure was recorded without timing relative to culture sampling, these data support prospective audit of carbapenem prescribing rather than demonstrating missed de-escalation.

## 1. Introduction

Healthcare-associated infections (HAIs) remain among the most frequent adverse events in acute care: in the 2022–2023 European point prevalence survey, 7.1% of patients in the sampled hospitals had at least one such infection, with country values between 3.1% and 13.8%, and the prevalence corrected for national validation studies was estimated at 8.0% (95% confidence interval [CI] 6.6–9.6%), corresponding to approximately 4.3 million patients affected each year in European Union/European Economic Area (EU/EEA) acute care hospitals [1]. Their clinical weight depends less on how often they occur than on the susceptibility of the organisms responsible: in microbiologically documented infections in that survey, 32.0% of the organisms included in the composite antimicrobial-resistance index were resistant [1]. Carbapenem-resistant Gram-negative bacteria occupy a particular position within that burden: the genera concerned are among those most frequently reported from healthcare-associated infections—*Klebsiella* spp. 11.7%, *Pseudomonas aeruginosa* 7.9% and *Acinetobacter* spp. 3.2% of all organisms in that survey—and carbapenem resistance removes the agents on which empirical therapy normally relies, which is why carbapenem-resistant Enterobacterales (CRE) and carbapenem-resistant *Acinetobacter baumannii* (CRAB) are classified as critical-priority pathogens, and carbapenem-resistant *P. aeruginosa* (CRPA) as high-priority, in the 2024 World Health Organisation (WHO) bacterial priority pathogens list [2]. Their prevalence varies widely across Europe: no carbapenem-resistant Enterobacterales isolate was reported by four countries, whereas Romania reported the highest national value, 42.9%, among the twenty-five countries contributing at least ten isolates with known susceptibility results [1].

Three kinds of data describe this problem, and they differ in what each can support. European surveillance networks report resistance proportions for invasive isolates or for organisms causing healthcare-associated infections, aggregated across hospitals of all types and sizes; they establish the ordering between countries [1,3]. Laboratory series, whether single-centre or multicentre, report clinical isolates of one species or from one specimen type and are generally not restricted to healthcare-associated infection, so that their denominators also contain community-onset isolates and, where active screening is performed, colonising ones [4,5,6,7]. Cohort studies that connect resistance to patient-level outcomes are largely confined to bloodstream infection or to intensive care, settings whose organism mix and severity distribution differ from those of a general infectious diseases ward [8,9,10]. Romanian participation in the European network was described in 2016 as involving a small number of laboratories reporting only carbapenem-resistant invasive *Klebsiella pneumoniae* and *Escherichia coli* from blood and cerebrospinal fluid [7]; the Romanian series available include laboratory reports restricted to carbapenem-resistant *K. pneumoniae* or *Acinetobacter baumannii* [4,5].

What these sources do not provide, individually or in combination, is the set of quantities on which empirical prescribing in a given centre actually depends: the proportion of Gram-negative healthcare-associated infections that are carbapenem-resistant, the number of antimicrobial classes that remain active within that resistant fraction, whether resistant and susceptible patients can be told apart using the variables a hospital routinely records, whether resistance status carries prognostic information beyond severity of illness, and how far carbapenem prescribing in the same cohort matches the microbiology that was documented. The third of these has been addressed directly by clinical prediction models, but the published models were derived in cohorts whose carbapenem-resistance prevalence ranged from 1.6% to 33% [11,12,13,14], and two of them predict colonisation at intensive care admission rather than infection [12,13], so their behaviour at the levels reported for south-eastern Europe has not been examined. Whether risk-based targeting retains operational value once resistance approaches saturation is therefore an empirical question rather than a premise.

The present study addresses these questions in a 24-month cohort of patients with microbiologically confirmed healthcare-associated infection at a tertiary infectious diseases hospital in western Romania. Its objectives are: (i) to quantify the prevalence of carbapenem resistance among Gram-negative isolates, overall, by species, across the CRE, CRAB and CRPA surveillance categories, and across the eight quarters of the study period; (ii) to quantify, within resistant isolates, the proportion designated extensively drug-resistant (XDR)—susceptible to no more than two of the antimicrobial classes tested—as an indicator of the treatment options remaining; (iii) to examine, as an exploratory analysis constrained by the small number of patients retaining a carbapenem-susceptible Gram-negative isolate, whether routinely recorded patient characteristics distinguish carbapenem-resistant from carbapenem-susceptible infection; (iv) to determine whether carbapenem resistance is associated with in-hospital mortality independently of intensive care exposure and comorbidity burden; and (v) to quantify the resulting stewardship gap, including carbapenem administration not supported by the documented microbiology, and to set the cohort’s reliance on last-resort agents against published pharmacovigilance signals for those agents. The same cohort has supported two further analyses by one of the authors, of infection recurrence, accepted for publication [15], and of antimicrobial treatment intensity and last-resort prescribing, currently under review; the present work differs from both in its primary outcome and in its unit of analysis, addressing the resistance phenotype of the infecting organisms rather than the clinical course or the treatment administered. Cohort descriptors and antimicrobial exposure frequencies, including the proportion of patients receiving a Reserve agent, necessarily recur across the three analyses and are reported here as context for the stewardship endpoint; no regression model and no resistance-related estimate is repeated.

## 2. Results

### 2.1. Cohort Characteristics and Stratification

Between 1 January 2024 and 31 December 2025, 315 patients with at least one microbiologically confirmed healthcare-associated infection were included, contributing 401 infection episodes and 485 isolates. Of these isolates, 477 were bacterial: 243 Gram-negative, 107 *Clostridioides difficile* and 127 other Gram-positive; the remaining eight were fungal or viral and carried none of the resistance markers analysed here. The 401 episodes comprised 138 lower respiratory tract infections (34.4%), 107 gastrointestinal infections (26.7%), 56 urinary tract infections and 56 episodes of sepsis (14.0% each), 32 skin and soft tissue infections (8.0%), eight venous catheter infections (2.0%) and four others (1.0%); each gastrointestinal episode yielded a single *C. difficile* isolate and no Gram-negative organism.

**Figure 1:**
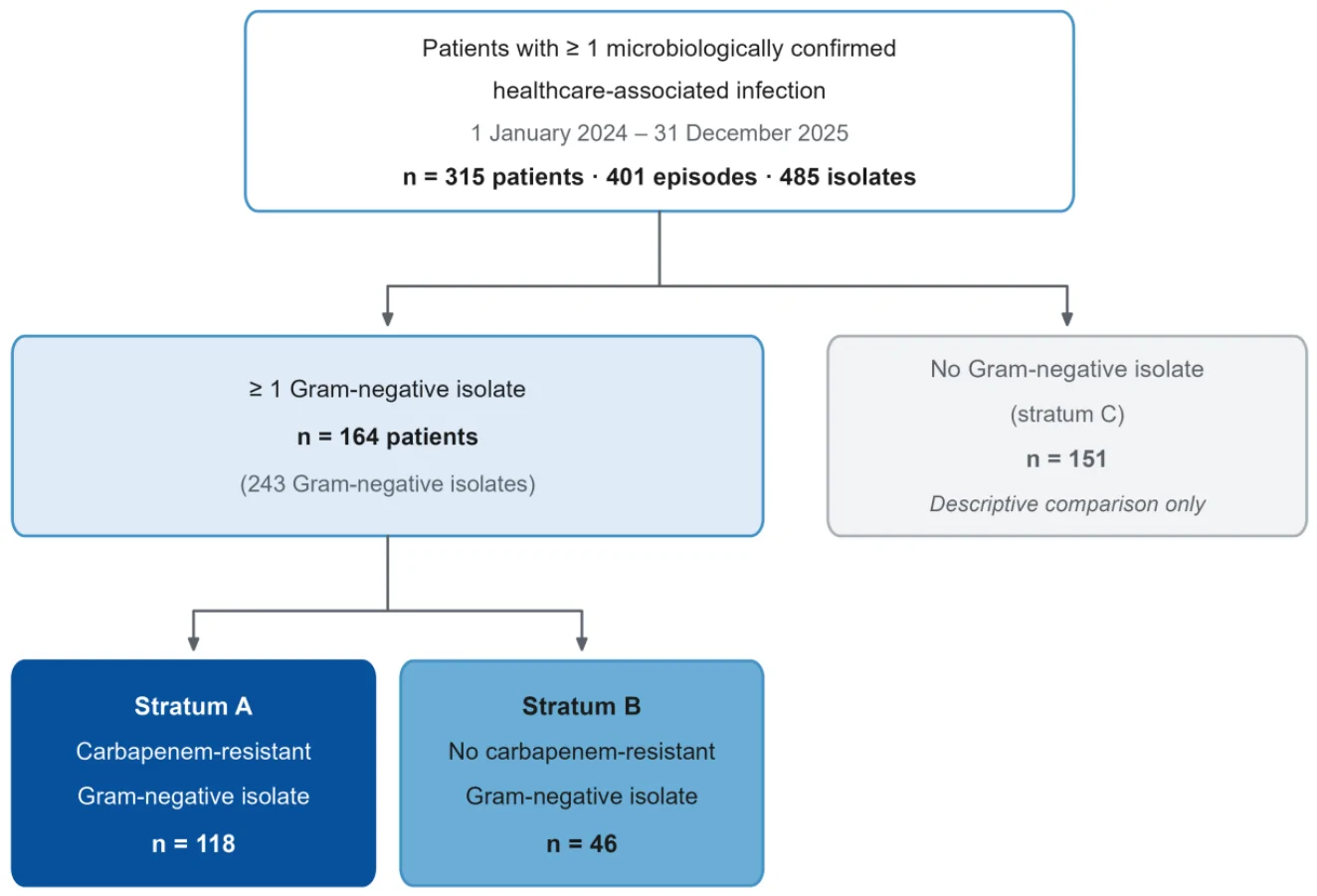
Flow of patients and isolates through the study. Strata A and B comprise patients with at least one Gram-negative isolate and are the two groups compared in the exploratory resistance analysis and in the mortality analysis; stratum C comprises patients without any Gram-negative isolate, for 89 of whom *Clostridioides difficile* was the only isolate recovered, and is presented descriptively only.

One hundred and sixty-four patients (52.1%) had at least one Gram-negative isolate. Within this group, 118 carried at least one carbapenem-resistant Gram-negative isolate (stratum A) and 46 did not (stratum B). The remaining 151 patients (47.9%) had no Gram-negative isolate at all (stratum C) and could not, by construction, meet the definition of the primary endpoint; for 89 of them *C. difficile* was the only isolate recovered. Table 1 presents the cohort by stratum, and Figure 1 shows the flow of patients and isolates through the study.

Overall, the cohort comprised 187 men (59.4%) with a median age of 69 years (interquartile range [IQR] 58–77); ninety-three patients (29.5%) were transferred to the intensive care unit (ICU), the median Charlson Comorbidity Index (CCI) was 5 (3–7), and in-hospital mortality was 38.7% (122/315). Strata A and B differed significantly only in intensive care exposure (ICU transfer 49.2% versus 30.4%, *p* = 0.046; ICU length of stay *p* = 0.024); age, sex, area of residence, comorbidity burden, length of stay, antibiotic exposure and mortality did not differ between them. Stratum C, shown for context, had markedly lower intensive care exposure (13.9%), antibiotic intensity and mortality (27.2%) than either Gram-negative stratum, which is why it is not used as a comparison group for resistance (Section 4.1).

Extended-spectrum β-lactamase (ESBL) production was recorded in 20 of the 36 carbapenem-susceptible Enterobacterales isolates (55.6%), including 10 of the 15 *Escherichia coli* isolates (66.7%). Because the registry records a single resistance marker per Gram-negative isolate, ESBL status is not available for carbapenem-resistant isolates (Section 4.4).

### 2.2. Carbapenem Resistance Among Gram-Negative Isolates

Of the 243 Gram-negative isolates, 162 (66.7%) were carbapenem-resistant. Within the three groups under European surveillance, 97 of 133 Enterobacterales (72.9%), 44 of 53 *Acinetobacter baumannii* (83.0%) and 17 of 51 *Pseudomonas aeruginosa* (33.3%) were carbapenem-resistant, corresponding to the CRE, CRAB and CRPA categories respectively and, at the patient level, to 77, 39 and 13 patients. The World Health Organisation ranks CRE and CRAB as critical-priority pathogens and CRPA as high-priority [2]. Among individual species (Table 2), the proportion reached 90.6% for *Klebsiella pneumoniae* (87 of 96) and 83.0% for *Acinetobacter baumannii* (44 of 53), against 33.3% for *Pseudomonas aeruginosa* (17 of 51); no *Escherichia coli* isolate was carbapenem-resistant (0 of 15). At the patient level, 118 of the 164 patients with a Gram-negative infection (72.0%) carried at least one carbapenem-resistant isolate.

Carbapenem resistance was rarely an isolated finding. Of the 162 carbapenem-resistant isolates, 150 (92.6%) were also designated XDR under the surveillance protocol, remaining susceptible to no more than two of the antimicrobial classes tested; only 12 (7.4%) retained susceptibility to three or more of the classes tested. This fraction was highest in the two dominant species—96.6% for *K. pneumoniae* and 95.5% for *A. baumannii*—and lowest for *P. aeruginosa* (64.7%). Expressed against all Gram-negative isolates rather than against the resistant fraction, 150 of 243 (61.7%) retained no more than two active classes. Table 2 shows both quantities by species. At the patient level, the designation was concordant with prescribing: 59 of the 109 patients with an XDR isolate (54.1%) received colistin, tigecycline or cefiderocol, against 2 of the 9 (22.2%) whose carbapenem-resistant isolates were not designated XDR and 7 of the 46 (15.2%) without a carbapenem-resistant isolate.

The corollary at the patient level is that the carbapenem-susceptible comparison group was small and, for the dominant species, close to absent: of 81 patients with a *K. pneumoniae* isolate, 72 carried a carbapenem-resistant one and 9 did not, and of 47 patients with *A. baumannii*, 39 carried a carbapenem-resistant isolate and 8 did not. Only *P. aeruginosa* retained a substantial susceptible group (13 of 45 patients carbapenem-resistant).

**Figure 2:**
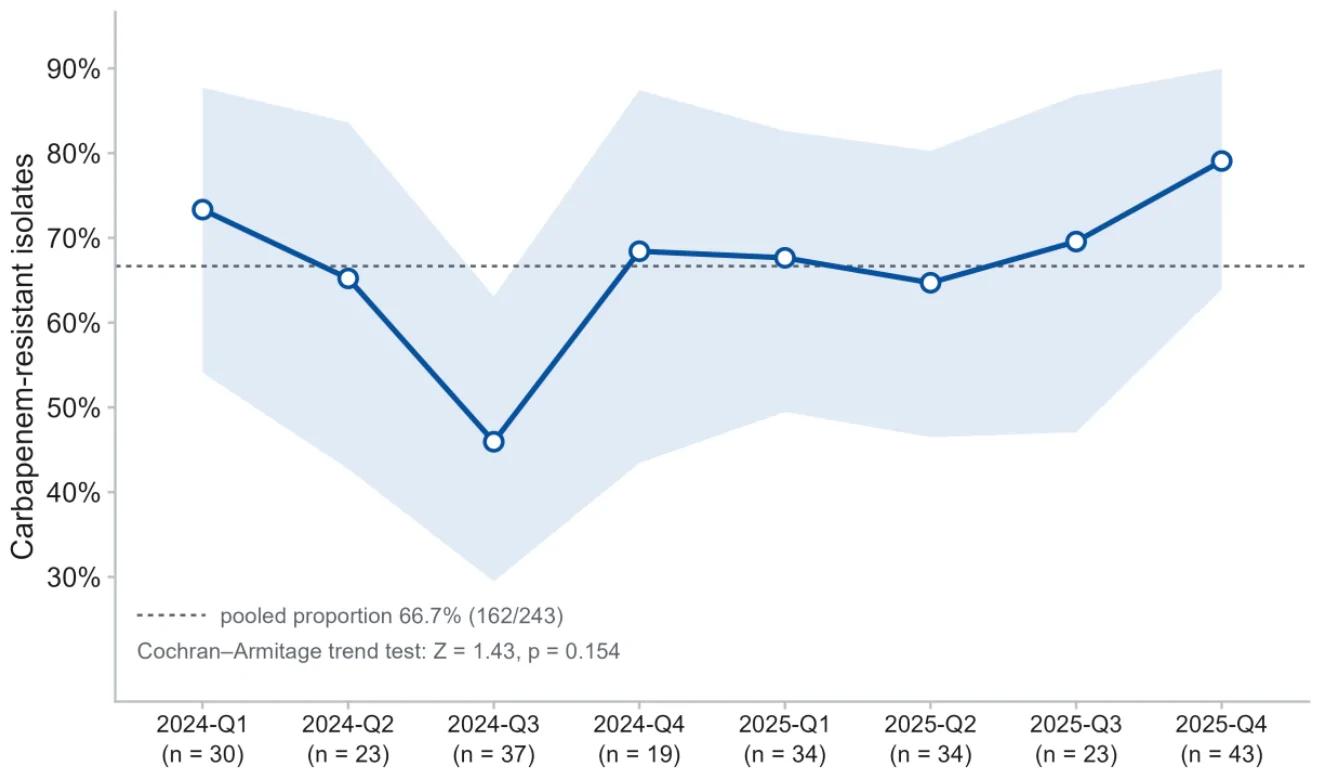
Quarterly proportion of carbapenem-resistant isolates among Gram-negative isolates, January 2024 to December 2025. Shaded band shows exact binomial 95% confidence intervals; n below each quarter is the number of Gram-negative isolates. The dashed line is the pooled proportion over the whole period.

The proportion of carbapenem-resistant isolates among Gram-negative isolates ranged from 45.9% to 79.1% across the eight quarters of the study period and exceeded 60% in seven of them (Figure 2). No statistically significant temporal trend was detected (Cochran–Armitage Z = 1.43, *p* = 0.154; odds ratio 1.09 per quarter, 95% CI 0.97–1.22), and between-quarter variation was compatible with chance (χ^2^ = 10.93, df = 7, *p* = 0.142); the corresponding tests for extensively drug-resistant isolates were likewise non-significant (Cochran–Armitage *p* = 0.339; χ^2^ *p* = 0.106).

Gram-negative isolates were recovered from 202 episodes, most often lower respiratory tract infections (129 isolates), followed by urinary tract infections (49), skin and soft-tissue infections (35), sepsis (20) and venous catheter infections (8) (Table 3). The specimens were sputum (50), tracheobronchial aspirates (46), bronchial aspirates (31) and pleural fluid (2) in respiratory infection, and urine (47), pressure-ulcer and purulent secretions (37), blood cultures (18) and venous catheter tips (8) at the other sites, with one isolate each from urethral secretion, a urinary catheter tip, conjunctival secretion and vaginal secretion. Among the four sites with at least 20 Gram-negative isolates, the proportion of carbapenem-resistant isolates ranged from 57.4% in lower respiratory tract infection to 90.0% in sepsis, and all 8 venous catheter isolates were carbapenem-resistant. This variation followed the species composition of each site rather than the site itself: *P. aeruginosa*, the species with the lowest resistance, accounted for 34 of the 129 respiratory isolates, whereas *K. pneumoniae* was carbapenem-resistant at every site, in 60 of its 61 isolates from non-respiratory infections.

### 2.3. Exploratory Comparison of Carbapenem-Resistant and Carbapenem-Susceptible Gram-Negative Infection

The 118 patients with a carbapenem-resistant Gram-negative isolate (stratum A) were compared with the 46 whose Gram-negative isolates were not carbapenem-resistant (stratum B); this is the only contrast available in the cohort that isolates resistance rather than organism type (Section 4.1). In the unadjusted comparison (Table 4), intensive care transfer was the only variable associated with resistance (49.2% versus 30.4%; crude odds ratio 2.21, 95% CI 1.07–4.56, *p* = 0.046). Receipt of three or more antibiotics showed the same direction without reaching significance (50.8% versus 34.8%; 1.94, 0.96–3.93, *p* = 0.093), while age, sex, area of residence, length of stay, comorbidity burden, and carbapenem exposure showed no association.

The adjusted model was restricted to three pre-specified exposures (Section 4.6). That the selection was not outcome-driven is illustrated by the retention of carbapenem exposure, whose unadjusted association was among the weakest of the eight candidates. None of the three was independently associated with resistance: intensive care transfer 1.86 (0.83–4.18, *p* = 0.132), carbapenem exposure 0.99 (0.45–2.19, *p* = 0.983) and three or more antibiotics 1.51 (0.66–3.42, *p* = 0.328). Discrimination was poor (area under the curve 0.618, 95% CI 0.529–0.707) and there was no collinearity (maximum variance inflation factor 1.33).

Two features of this analysis bound its interpretation. The single significant unadjusted association arose among eight comparisons made without adjustment for multiplicity, a yield consistent with chance alone; and it did not persist after adjustment for antibiotic exposure, which is consistent with treatment intensity reflecting, rather than preceding, the resistance of the infecting organism: antibiotic exposure is recorded for the hospitalisation as a whole, without timing relative to culture sampling, and cannot be read as antecedent to resistance. No patient-level characteristic recorded in this registry distinguished carbapenem-resistant from carbapenem-susceptible Gram-negative infection with useful precision.

### 2.4. In-Hospital Mortality

**Figure 3:**
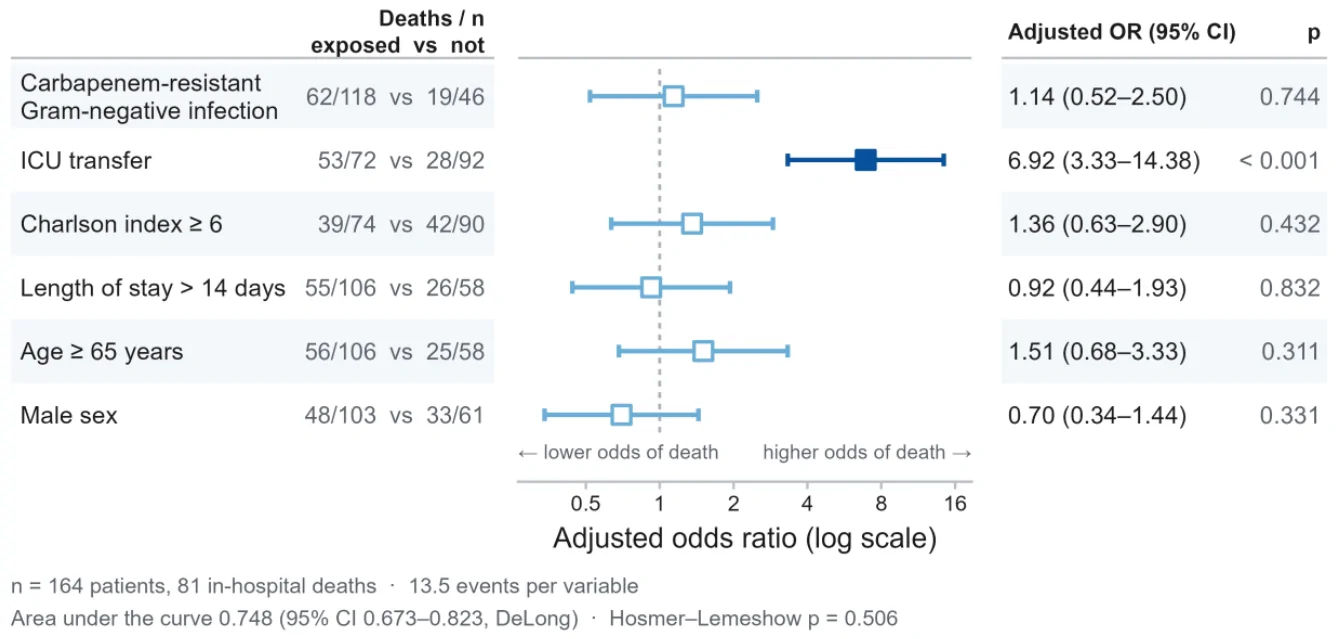
Multivariable logistic regression for in-hospital mortality among patients with a Gram-negative healthcare-associated infection (n = 164). Squares are adjusted odds ratios, and horizontal lines are their 95% confidence intervals, plotted on a logarithmic scale; the filled square marks the only interval that excludes 1. Deaths/n are crude counts of in-hospital death in each exposure group, shown for orientation only; the odds ratios are mutually adjusted for all six covariates listed. The analysis is restricted to patients with a Gram-negative isolate so that the comparison group consists of patients whose Gram-negative infection was carbapenem-susceptible rather than patients with a different infection type. CI, confidence interval; ICU, intensive care unit; OR, odds ratio.

Among the 164 patients with a Gram-negative healthcare-associated infection, 81 (49.4%) died in the hospital: 62 of 118 (52.5%) with a carbapenem-resistant isolate and 19 of 46 (41.3%) without (crude odds ratio 1.57, 95% CI 0.79–3.13). After adjustment for intensive care transfer, comorbidity burden, length of stay, age and sex (Figure 3), carbapenem resistance was not associated with in-hospital death (adjusted odds ratio 1.14, 95% CI 0.52–2.50, *p* = 0.744). Intensive care transfer was the only independent correlate of mortality (6.92, 3.33–14.38, *p* < 0.001); the Charlson index, length of stay, age and sex were not. The model gave acceptable discrimination (area under the curve 0.748, 95% CI 0.673–0.823) with no evidence of poor calibration (Hosmer–Lemeshow *p* = 0.506) and 13.5 events per variable.

### 2.5. Antimicrobial Stewardship and Exposure to Last-Resort Agents

Ninety-two patients (29.2%) received at least one carbapenem. Five (5.4%) had an ESBL-producing, carbapenem-susceptible Enterobacterales isolate and no carbapenem-resistant one, for which carbapenem therapy is guideline-concordant. Fifty-four (58.7%) had a carbapenem-resistant Gram-negative isolate, against which carbapenems are not active; 34 of these 54 also received colistin, tigecycline or cefiderocol, and one received a carbapenem as the only antibiotic. Twelve patients (13.0%) had a Gram-negative isolate that was neither carbapenem-resistant nor ESBL-producing, and 21 (22.8%) had no Gram-negative isolate at all (Table 5).

One hundred and two patients (32.4%) received at least one Reserve antibiotic under the WHO Access, Watch, Reserve (AWaRe) classification and 93 (29.5%) received three or more antibiotics. Forty-nine patients (15.6%) were both in the high comorbidity category and exposed to a Reserve agent.

Table 6 sets the cohort’s exposure to last-resort agents against published EudraVigilance disproportionality signals for drug resistance and drug ineffectiveness adverse-reaction terms, reproduced from Vintila et al. [16]. These reporting odds ratios are derived from the European spontaneous-reporting database and are not observations of the present cohort. Colistin, received by 23.2% of patients, carried the strongest resistance-related signal, with a reporting odds ratio of 16.91 (95% CI 12.57–22.74) against moxifloxacin for drug resistance terms and 24% of such reports fatal; for meropenem and linezolid the values shown are for drug ineffectiveness terms. Taken together with the concentration of Reserve prescribing described above, these external signals support therapeutic drug monitoring and early de-escalation in the high-comorbidity subgroup exposed to Reserve agents.

## 3. Discussion

Over 24 months of structured surveillance, 243 Gram-negative isolates were recovered from 202 of the 401 microbiologically confirmed healthcare-associated infection episodes, and 162 of them (66.7%) were carbapenem-resistant, affecting 118 of the 164 patients (72.0%). Resistance was concentrated in the two dominant species—87 of 96 *Klebsiella pneumoniae* isolates (90.6%) and 44 of 53 *Acinetobacter baumannii* isolates (83.0%)—against 17 of 51 *Pseudomonas aeruginosa* (33.3%) and none of 15 *Escherichia coli*. Resistance was present at every infection site, and its variation between sites followed the species composition of each rather than the site itself (Table 3). Within the resistant fraction, 150 of 162 isolates (92.6%) were designated XDR, susceptible to no more than two of the antimicrobial classes tested, leaving only 12 with three or more. Two further findings were negative. None of the eight routinely recorded patient characteristics distinguished carbapenem-resistant from carbapenem-susceptible Gram-negative infection after adjustment, and discrimination was poor (area under the curve 0.618). In-hospital death was independently associated with intensive care transfer but not with carbapenem resistance.

Four features of the data indicate that these proportions reflect the organisms recovered rather than the way resistance was recorded. First, isolate-level and patient-level estimates almost coincide—90.6% against 88.9% (72 of 81 patients) for *K. pneumoniae*, and 83.0% against 83.0% (39 of 47 patients) for *A. baumannii*—so repeated isolates from the same patients do not drive the figures. Second, the recorded phenotypes discriminate between resistance mechanisms within a single species rather than being applied uniformly: no *E. coli* isolate was carbapenem-resistant, yet 10 of 15 (66.7%) produced an extended-spectrum β-lactamase, a pattern that indiscriminate labelling would not generate. The species gradient points the same way, with resistance in *P. aeruginosa* approximately one-third of that in *K. pneumoniae*. Third, the stratum without any Gram-negative isolate behaved as its composition predicts, with intensive care exposure of 13.9% and mortality of 27.2%, consistent with *Clostridioides difficile* being the only organism recovered in 89 of those 151 patients. Fourth, variables with no mechanistic reason to track resistance did not do so: age, sex, area of residence, comorbidity burden and length of stay were all unassociated in the exploratory comparison, which is what a dataset free of a generalised recording artefact should show.

Compared with European surveillance using the same denominator—organisms causing healthcare-associated infections—the excess resistance in this cohort is species-specific rather than uniform. In the 2022–2023 EU/EEA point prevalence survey, carbapenem resistance was reported in 82.9% of *A. baumannii* isolates from healthcare-associated infections, an aggregate over the only ten countries that contributed at least ten isolates each (429 isolates; national values 61.7% in Croatia to 100% in Cyprus), and in 29.7% of *P. aeruginosa* isolates across twenty countries (1208 isolates; 0% in Finland to 63.6% in Bulgaria) [1]. The corresponding values here, 83.0% and 33.3%, therefore fall within the range of the European countries that report these organisms in sufficient numbers, rather than above it. For the Enterobacterales the position differs: the same survey reports 9.3% across the EU/EEA and 25.1% for *K. pneumoniae*, and Romania has the highest national value for Enterobacterales among the twenty-five reporting countries at 42.9%, with 63.3% for *K. pneumoniae*, third behind Bulgaria (70.4%) and Greece (63.6%) among the twenty countries reporting that species [1]. The values here, 97 of 133 (72.9%) and 90.6%, exceed the national ones by roughly 30 percentage points in both cases, so that the gradient has a national and a centre component rather than a single unexplained excess. Nor is the excess uniform within the Enterobacterales. A regional point prevalence survey of eighteen Italian acute care hospitals, conducted in 2016 under the ECDC protocol and therefore on the same denominator, reported carbapenem resistance in 24 of 90 *Enterobacteriaceae* isolates from healthcare-associated infections (26.7%); that aggregate combined 22 of 34 *Klebsiella* spp. (64.7%) with 1 of 37 *Escherichia coli* (2.7%) [17]. The present cohort reproduces that ordering more sharply—90.6% for *K. pneumoniae* against none of 15 *E. coli* isolates—which locates the excess within *Klebsiella* rather than across the order, and makes the genus composition of the denominator a condition of any comparison drawn at the level of Enterobacterales. Two qualifications apply to any such comparison. The survey is a one-day cross-sectional exercise, whereas the present data are cumulative over 24 months; and the report itself notes that overall European percentages are strongly influenced by the few countries contributing large numbers of resistant isolates, which is a reason to read these figures as an ordering rather than as a calibration.

Cohorts from the same region show the same asymmetry between Enterobacterales and non-fermenting organisms, with the present centre positioned differently in each arm. A Serbian national point prevalence survey reported carbapenem resistance in 103 of 128 non-fermentative Gram-negative bacilli (80.5%) but in 50 of 139 Enterobacterales (35.9%) [18], against 61 of 104 (58.7%) for *A. baumannii* and *P. aeruginosa* combined and 97 of 133 (72.9%) for Enterobacterales here; the non-fermenter groupings are not strictly equivalent, since the Serbian category is not defined in the source and may include species with intrinsic carbapenem resistance, whereas the present figure is restricted to the two genera under European surveillance. A pooled analysis of intensive care isolates from Latin America and the Caribbean reported the same ordering, 72.58% for *A. baumannii* against 37.48% for *K. pneumoniae*, although with substantial between-study heterogeneity [19]. Comparison with a Romanian infectious diseases hospital is informative precisely because it is discordant: 217 of 521 *K. pneumoniae* isolates (41.6%) were carbapenem-resistant there in 2024, but that denominator comprises all non-repetitive clinical isolates of the species at a hospital whose mean length of stay is about six days, and urine accounted for 59.0% of the resistant strains recovered, whereas the present denominator is restricted to healthcare-associated infection in a cohort with a median stay of 18 days [4]. Within Romania the same species-level figure varies with case-mix: a cardiovascular and transplant centre reported carbapenem resistance of 55% in *K. pneumoniae* on its cumulative antibiogram across 139 healthcare-associated infections [20], against 90.6% here. A further Romanian series illustrates how far the denominator governs such figures: among 406 isolates already classified as multidrug-resistant, carbapenem resistance exceeded 90% by the end of the study period, a proportion conditional on prior selection for resistance and therefore not comparable with proportions computed over all isolates of a species [21]. A five-year Italian series with a closely similar denominator structure—154 of 402 Gram-negative infection isolates (38.3%) from 382 patients—shows the same contrast between a general hospital and a referral centre for infection [22]. Taken together, these comparisons indicate that the distinguishing feature of the present cohort is the Enterobacterales fraction rather than the overall level of resistance.

The proportion of resistant isolates retaining no more than two active antimicrobial classes, 92.6%, has a more direct clinical consequence than prevalence, but it is also the quantity least comparable across studies, because the criterion has been operationalised in at least three ways. Applying non-susceptibility to all but two or fewer antimicrobial categories as tabulated by Magiorakos et al. [23], a Greek tertiary hospital classified 791 of 1142 carbapenem-resistant bloodstream isolates (69.3%) as extensively drug-resistant [6], whereas a selected Bulgarian multicentre series of 226 carbapenem-non-susceptible *A. baumannii* isolates classified only 28 (12.4%) [24]. The Bulgarian figure illustrates how far the result depends on what was tested rather than on what was resistant: tigecycline and ampicillin–sulbactam remained active in 77.9% and 58.4% of those isolates respectively, and their presence on the panel was sufficient to keep most isolates above the threshold. Applying the same framework to 340 non-duplicate carbapenem-non-susceptible *K. pneumoniae* isolates, a Romanian tertiary centre classified 192 (56.5%) as extensively drug-resistant [25], against 84 of the 87 carbapenem-resistant *K. pneumoniae* isolates here (96.6%). A Romanian multicentre series applying the criterion at the level of pharmacological classes reported 108 of 118 *A. baumannii* isolates (91.52%) as extensively drug-resistant [5], against 42 of 53 (79.2%) here, both evaluating all isolates of the species rather than the resistant fraction alone. In the present study the threshold was evaluated against the antimicrobial classes actually tested for each isolate rather than against the fixed, organism-specific category list of the framework, which requires that isolates be tested against all or nearly all agents in all categories and that selective reporting be avoided [23]; because a routine susceptibility panel comprises fewer classes than the complete category list, the proportion designated extensively drug-resistant is expected to be higher than under a strict category-based application, whereas the counting of intermediate results as retained classes and the testing of colistin by VITEK 2 in some isolates act in the opposite direction (Section Limitations and Section 4.4); the composition of the panel is not retained in the registry, so the net difference cannot be quantified. The figure is accordingly reported as surveillance-assigned and is not interchangeable with estimates obtained by applying the framework to a complete category list. Application of the framework is itself heterogeneous: extensive drug resistance has been defined by antimicrobial categories [20,25], by pharmacological classes [5,26] and by the number of active agents [4], renderings that are not interchangeable and that are seldom flagged as differing. Whatever framework is applied, the underlying observation stands: under the local rule, only 12 of 162 resistant isolates retained three or more of the classes tested; which classes these were is not recorded, so the margin for empirical error cannot be reconstructed but was evidently narrow. The designation was nonetheless concordant with the use of last-resort agents (Section 2.2); without timing of administration, this concordance is descriptive and does not validate the designation against a reference classification.

Carbapenem resistance was stable over time: it exceeded 60% in seven of the eight quarters, never fell below 45.9%, and showed no significant trend across the period (Cochran–Armitage *p* = 0.154). The prevalence reported here was therefore sustained across the 24 months rather than produced by an epidemic episode within them, although the study was not powered to exclude a modest increase. That no patient-level characteristic separated resistant from susceptible infection appears to contradict a substantial risk-factor literature, and the discrepancy is itself informative. In a meta-analysis of 85 studies and 22,396 patients, prior antibiotic exposure was associated with acquisition of carbapenem-, extensively drug- and pandrug-resistant *A. baumannii* and *P. aeruginosa* both when cases were compared with susceptible cases (odds ratio 2.06) and when compared with uninfected patients (4.16) [27], and a case-case–control study reported more than eighteen-fold higher odds of antibiotic exposure in the 90 days before admission in carbapenem-resistant than in carbapenem-susceptible Enterobacterales infection [28]. Three considerations reconcile these with the present null. First, exposure in those studies is antecedent by construction, whereas antibiotic administration in this registry is recorded for the hospitalisation as a whole, without attribution to a specific infection or timing relative to culture sampling, and cannot be interpreted as preceding resistance; the two designs do not measure the same quantity. Second, the only comparison available here is between resistant and susceptible cases, a control structure shown to alter effect estimates for antibiotic exposure more than fourfold in the same patients, from an odds ratio of 27.12 (95% CI 13.91–52.90) to 6.34 (3.66–11.00) for imipenem [29]; under a design known to distort estimates of antibiotic exposure, no association emerged. Third, stratification requires a susceptible comparison group, and for the two species carrying most of the burden that group has nearly disappeared, with 9 patients retaining a carbapenem-susceptible *K. pneumoniae* and 8 a carbapenem-susceptible *A. baumannii*. The consequence is best expressed through the conditional probabilities rather than the *p*-value: 58 of the 72 patients transferred to intensive care carried a carbapenem-resistant isolate (80.6%), against 60 of the 92 who were not (65.2%). Both lie far above any threshold at which a clinician would withhold cover for carbapenem-resistant organisms, so knowing the variable does not change the empirical decision; and because intensive care transfer was the strongest of the eight examined, the argument applies a fortiori to the remainder.

Published prediction models are consistent with this reading. Areas under the curve of 0.846, 0.825, 0.80 and 0.80 were achieved in derivation cohorts whose carbapenem-resistance prevalence was 1.6%, 12%, 20.0% and 33% respectively [11,12,13,14], the highest of these less than half the prevalence reported here, and the best-discriminating model had a sensitivity and a positive predictive value of 30% [11], which illustrates that discrimination and clinical usefulness need not coincide where prevalence is extreme. Two of the four predict colonisation at intensive care admission rather than infection [12,13], an outcome that the national surveillance system does not record and that could not be reproduced here. The same pattern appears in surveillance data: across 18,412 invasive *Acinetobacter* spp. isolates from 30 European countries, among which regional proportions of carbapenem non-susceptibility ranged from 2.8% in Northern to 75.5% in Southern Europe, intensive care admission was independently associated with resistance [3], the contrast between settings supplying variation that a single saturated setting does not contain. A Greek secondary-care cohort reached the same negative conclusion within one setting, finding no association between multidrug- or extensively drug-resistant bacteraemia and sex, age, comorbidity, pre-culture length of stay, or prior intensive care stay [8]. Stratifiability therefore appears to depend on the resistance level of the setting rather than on the choice of method, and no increase in sample size would render stratifiable, in a way that alters management, a population in which 90.6% of *K. pneumoniae* and 83.0% of *A. baumannii* isolates are already resistant.

In-hospital mortality among patients with a Gram-negative healthcare-associated infection was 81 of 164 (49.4%) but was not independently associated with carbapenem resistance. One objection deserves explicit treatment: if resistant infection causes the deterioration that leads to intensive care transfer, then adjusting for transfer removes part of the effect of interest. The unadjusted estimate, however, is also null (odds ratio 1.57, 95% CI 0.79–3.13), so the conclusion does not rest on the adjustment. A crude gradient that does not survive adjustment for severity recurs in comparable cohorts: mortality did not differ between 30 patients with carbapenem-resistant and 66 with carbapenem-susceptible *A. baumannii* bacteraemia in a Greek intensive care unit (43.3% against 46.9%), where severity of organ failure rather than resistance predicted death (odds ratio 1.42, 95% CI 1.20–1.67) [30]; and case fatality of 22%, 35% and 63% across non-multidrug-resistant, multidrug-resistant and extensively drug-resistant bacteraemia corresponded to a non-significant cause-specific hazard ratio for 14-day death of 1.61 (0.45–5.78) after adjustment for age, intensive care stay and comorbidity [8]. A Romanian cardiovascular and transplant centre reached the same conclusion in 139 healthcare-associated infections, where intensive care admission and treatment with more than three antibiotics, rather than resistance status, were independently associated with mortality [20]. A prospective multicentre study of 632 *P. aeruginosa* bacteraemia episodes suggests a mechanism: the effect of carbapenem resistance on mortality interacted significantly with comorbidity, the adjusted hazard ratio at day 30 falling from 9.9 (3.3–29.4) at a Charlson index of 0 to 2.6 (0.8–8.0) at a Charlson index of 5, and becoming statistically significant only from the fifth day after onset [31]. The median Charlson index in the present cohort was 5.

The literature is heterogeneous rather than uniformly positive: a meta-analysis of 16 studies of *A. baumannii* infection reported a pooled adjusted odds ratio of 2.49 (1.61–3.84) across the ten that reported adjusted estimates, which fell to 1.77 (1.22–2.55) when four studies reporting no univariate association were reinstated with an assumed odds ratio of 1, and its constituent adjusted estimates include 0.73 (0.14–3.84) and 1.03 (0.43–2.20) [32]. Studies with stronger designs do report an association, including an adjusted subdistribution hazard ratio of 1.75 (1.04–2.94) from competing-risk models in 297 patients with *Enterobacteriaceae* bloodstream infection [10], and, relative to carbapenem-susceptible Gram-negative bloodstream infection and after adjustment for Sequential Organ Failure Assessment (SOFA) score, adjusted odds ratios for 30-day mortality of 2.65 (1.52–4.61) for carbapenem-resistant *A. baumannii* and 5.86 (2.72–12.76) for metallo-β-lactamase-producing Enterobacterales [33]. In a national prevalent cohort of 8247 inpatients in 37 Greek acute care hospitals, followed to 90 days with correction for confounding and time-dependent bias, carbapenem-resistant Enterobacterales and carbapenem-resistant *Acinetobacter baumannii* infections carried adjusted hazard ratios for death of 1.9 (95% CI 1.1–3.2) and 1.9 (1.1–3.0) respectively [34]; that cohort measured 90-day rather than in-hospital mortality and applied a design the present analysis could not. The present estimate is therefore best read as lying in the lower part of a heterogeneous distribution rather than as a refutation of it.

If resistance is neither predictable at the patient level nor an independent determinant of death in this population, the operational lever shifts from selecting patients to auditing prescribing. Among the 92 carbapenem recipients, three groups carry a distinct operational meaning. Fifty-four had a carbapenem-resistant isolate, against which a carbapenem is not active; 34 of them also received colistin, tigecycline or cefiderocol and only one received a carbapenem as the sole antibiotic, a pattern consistent with empirical carbapenem therapy subsequently supplemented or replaced by an active agent, which the registry cannot confirm without timing data. For twelve patients (13.0%) a narrower alternative was already documented, and these are the concrete de-escalation target. A further twenty-one (22.8%) received a carbapenem without any Gram-negative isolate being recovered, which raises a question of empirical indication rather than of de-escalation and cannot be adjudicated without clinical review. That such a gap persists where two-thirds of Gram-negative isolates are resistant is unsurprising given the converse observation elsewhere: empirical therapy targeting Gram-negative pathogens was inappropriate in 77 of 130 patients (59.2%) across 20 Italian intensive care units in which 19.2% of Enterobacterales infections were carbapenem-resistant [9], and carbapenems had been administered before detection of a carbapenem-resistant organism in 62.2% of 119 affected patients at a German university centre [35].

The reliance on last-resort agents that follows from the depth of resistance carries its own cost: nearly a third of the cohort received at least one Reserve antibiotic under the WHO AWaRe classification [36] and 73 patients (23.2%) received colistin, in a country where another centre reported colistin susceptibility of 16.5% among New Delhi metallo-β-lactamase (NDM)- and oxacillinase-48 (OXA-48)-co-producing isolates and an increase in colistin consumption from 11,787 to 21,870 therapeutic units between 2019 and 2024 [4], and where the prevalence of polymyxin or tigecycline use correlates with the proportion of carbapenem-resistant Enterobacterales at the country level (Spearman’s rho 0.67, *p* < 0.001) [1]. Published disproportionality analyses of the European spontaneous-reporting database place colistin above the other last-resort agents examined for adverse-reaction terms denoting drug resistance and drug ineffectiveness (Table 6) [16]; since these signals are external to the present cohort and subject to reporting bias, they support therapeutic drug monitoring and early de-escalation in the subgroup exposed to Reserve agents as a patient-safety measure rather than establishing harm in these patients. One limit of any such recommendation should be stated: without molecular typing, clonal transmission cannot be distinguished from independent selection under antibiotic pressure—documented nosocomial transmission accounted for 24.4% of cases in the German cohort—and the two have different remedies, infection prevention in the first case and prescribing in the second.

### Limitations

Resistance was ascertained from phenotype flags recorded in the surveillance registry rather than from category-level antibiograms, which were not retained in the registry. Consequently, the proportion of isolates susceptible to individual antimicrobial agents cannot be reported, and the XDR designation, although it rests on the antibiogram of each isolate, cannot be accompanied by the underlying susceptibility profiles. The multidrug-resistance classification of the European Centre for Disease Prevention and Control/Centres for Disease Control and Prevention (ECDC/CDC) consensus could therefore not be derived; the extensively drug-resistant designation was assigned against the classes tested for each isolate rather than against the fixed category list of that framework, which raises the proportion relative to a strict category-based application, while the treatment of an intermediate result as a retained class acts in the opposite direction; neither the agents tested nor their individual results were retained in the surveillance registry, so the designation cannot be recomputed and the net difference cannot be quantified. The extensively drug-resistant category is defined internationally [23], but it is not among the antimicrobial resistance markers collected under the national surveillance methodology, which records susceptibility for a fixed set of organism-drug combinations [37]; the field is therefore assigned under the hospital surveillance reporting protocol by the reporting physicians and the infection prevention and control team, and the rule by which it is applied—evaluation against the classes tested rather than against the organism-specific category lists of the framework—is set locally. The equivalence of XDR designation and carbapenem resistance reflects reporting practice at this hospital rather than a formal rule; a carbapenem-susceptible isolate designated XDR would have been counted as carbapenem-resistant, although none is expected under that practice. The exclusion of intrinsic resistance, which the reporting protocol applies as the framework requires [23], could not be verified retrospectively, as illustrated by the three *Stenotrophomonas maltophilia* isolates, only one of which carries a carbapenem-resistance flag although the species is intrinsically carbapenem-resistant. Breakpoint tables were revised between the two study years (European Committee on Antimicrobial Susceptibility Testing [EUCAST] v14.0 to v15.0), which may affect temporal comparability. ESBL production is not recorded for carbapenem-resistant isolates, because the registry records a single marker per Gram-negative isolate, and AmpC production was not tested. Colistin susceptibility was determined by broth microdilution or by VITEK 2; broth microdilution is the only reference method for colistin and automated systems may fail to detect resistance, and because polymyxins were counted as a retained class when colistin susceptibility had been demonstrated, isolates tested by VITEK 2 may have been designated XDR less often than under broth microdilution. Carbapenemase production was investigated only in selected isolates, and neither these results nor genotypic data are recorded in the registry; this is more than a descriptive gap: mortality attributable to carbapenem resistance is mechanism-specific, ranging from 5% for *Klebsiella pneumoniae* carbapenemase-producing to 35% for metallo-β-lactamase-producing Enterobacterales [33], so an aggregate resistance endpoint averages over effects of very different magnitude, and the direction of that averaging cannot be established from the present data. National data indicate which mechanisms are likely to be involved: OXA-48-like and NDM carbapenemases, frequently co-produced, have been reported in carbapenem-resistant *K. pneumoniae* from Romanian hospitals [4,7], and OXA-23-like and OXA-24/40-like genes in 91.5% and 74.6%, respectively, of 118 clinical *A. baumannii* isolates in a Romanian multicentre series [5]; the mechanism mix of the present isolates may resemble this pattern, but this cannot be confirmed.

The variables examined were those recorded in routine surveillance. Infection site and specimen are recorded per episode rather than per isolate, and specimen quality is not graded; one fifth of Gram-negative isolates came from sputum, in which the distinction between infection and colonisation rests on the surveillance case definition. Excluding the 50 sputum isolates, the carbapenem-resistant proportion was 69.9% (135 of 193), so isolates from the specimen in which colonisation is most difficult to exclude did not inflate the estimate. Prior healthcare exposure, transfer from another facility, residence in long-term care, exposure to invasive devices and previous colonisation status are absent, and transfer from another hospital, intensive care admission within the preceding year and antibiotic exposure within three months have each been identified as predictors of carbapenem-resistant Enterobacterales acquisition [38]. The absence of stratification therefore applies to routinely recorded data rather than to resistance risk in principle. Antibiotic exposures are recorded at the patient level as the agents administered during the hospitalisation, cannot be attributed to a specific infection, community-onset or healthcare-associated, or to a specific episode, and carry no timing relative to culture sampling, so they can be interpreted neither as antecedent to resistance nor as evidence of missed de-escalation. Colonisation status is absent from the registry, so the case–case–control design [39], applied elsewhere where such data exist [28], could not be used here; the consequences for the exposure estimates are set out in Section 4.1.

Mortality was assessed in hospital and modelled by logistic regression without treating discharge alive as a competing risk, an approach that has yielded larger estimates elsewhere [10]. Patients who die early also have less opportunity both to acquire a resistant organism and to have one recovered in culture, and this survivorship effect would bias the resistance–mortality association towards the null, so the null reported here is conservative in that respect. Comorbidity burden was captured by the CCI, but acute physiological severity was not: it enters the mortality model only through intensive care transfer, and neither a score such as SOFA nor any measure of device exposure is recorded in the registry, so residual confounding of the resistance–mortality association by acute illness severity cannot be excluded. Finally, the exploratory comparison included only 46 patients in the smaller stratum and is underpowered to detect modest resistance-specific associations; the absence of statistically significant predictors should be read as failure to detect rather than as evidence of absence, whereas the near absence of a susceptible comparison group at the species level is a finding rather than a limitation of the analysis.

## 4. Materials and Methods

### 4.1. Study Design and Setting

Single-centre retrospective cohort study at the “Dr. Victor Babeș” Clinical Hospital of Infectious Diseases and Pneumophthisiology, Timișoara, Romania, a tertiary-care university-affiliated hospital specialising in infectious diseases and pneumophthisiology, over 24 consecutive months (1 January 2024–31 December 2025).

All patients in this cohort met the inclusion criterion of at least one microbiologically confirmed healthcare-associated infection; the study is therefore case-only with respect to infection, and no uninfected control group could be constituted from the surveillance registry. The case–case–control design proposed for risk-factor studies of antimicrobial resistance, in which cases infected with the resistant organism and cases infected with its susceptible counterpart are each compared with controls drawn from the source population [39], could consequently not be implemented, since colonisation is not reportable under the national surveillance system and patients without the organism of interest are therefore not enumerated; the design has been applied where such data are available [28]. Patients whose healthcare-associated infection involved organisms other than Gram-negative bacteria constitute a third case group rather than a control group, and comparisons involving them are subject to severity-of-illness bias. The comparison that remains available—resistant cases against susceptible cases of the same organism group—corresponds to the control structure shown to distort estimates of antimicrobial exposure relative to a control group drawn from the source population [29]; estimates of antibiotic exposure obtained under the present design are to be read in that light.

The cohort was accordingly stratified into three strata: patients with a carbapenem-resistant Gram-negative isolate (n = 118), patients with a Gram-negative isolate that was not carbapenem-resistant (n = 46), and patients without any Gram-negative isolate (n = 151). The only comparison that isolates resistance rather than organism type is that between the first two strata, and it is reported as an exploratory analysis. Comparisons involving the third stratum are presented descriptively and are not interpreted as risk factors for resistance. Species-stratified comparison of resistant with susceptible isolates, the conventional design in this literature, was not feasible for the two dominant species, since only 9 patients with *K. pneumoniae* and 8 with *A. baumannii* carried a carbapenem-susceptible isolate.

### 4.2. Study Population

Inclusion criteria:

- patients admitted during the study period (seven were children aged 1 to 12 years, two of whom had a Gram-negative isolate);
- ≥1 microbiologically confirmed HAI. Under the European surveillance case definitions adopted in the national methodology (Commission Implementing Decision (EU) 2018/945), an infection is associated with the current admission when symptom onset falls on day 3 or later, the day of admission being day 1, or when it follows surgery or insertion of an invasive device performed on day 1 or day 2. In the present registry the criterion was applied to the date of collection of the qualifying specimen, which was on or after day 3 of admission in all included episodes; site-specific infection criteria followed the CDC National Healthcare Safety Network (NHSN) definitions [40];
- complete data for the primary outcome and key covariates.

The final cohort comprised 315 patients (251 single-episode, 64 recurrent), contributing 401 HAI episodes and 485 isolates. Only episodes meeting these case definitions are reportable under the national healthcare-associated infection surveillance system; colonisation is not reportable and is not recorded in the registry. Patients with only fungal or viral isolates were retained in cohort descriptives but, by definition, contributed no bacterial resistance markers. Infection site and specimen type are recorded per episode; in episodes yielding more than one isolate, all isolates were attributed to the specimen recorded for that episode.

### 4.3. Database Structure

Data were organised in a three-level relational database (patient/episode/isolate), reconciled through the unique patient identifier and HAI-episode number.

### 4.4. Phenotypic Resistance Definitions

Resistance phenotypes were retrieved from the hospital infection-control surveillance registry as discrete binary fields, of which three apply to Gram-negative organisms: carbapenem-resistant (CARBA), extended-spectrum β-lactamase (ESBL) and extensively drug-resistant (XDR). Of these, the CARBA field corresponds to the antimicrobial resistance markers collected under the national healthcare-associated infection surveillance methodology, which records susceptibility to imipenem, meropenem and doripenem for *Enterobacteriaceae*, *Pseudomonas aeruginosa* and *Acinetobacter* spp. and is aligned with the European Antimicrobial Resistance Surveillance Network (EARS-Net) and Healthcare-Associated Infections Surveillance Network (HAI-Net) protocols [37]; the ESBL and XDR fields are additional to that set. The CARBA and ESBL markers are determined by the laboratory, whereas the XDR designation is assigned by the reporting physicians and the infection prevention and control team, as described below. Isolates were identified by Gram staining, colony morphology, conventional biochemical testing (oxidase, citrate, urease, indole, motility and ONPG) and the API 10S gallery (bioMérieux, Marcy-l’Étoile, France), supplemented for selected isolates by the VITEK 2 system (bioMérieux) or by matrix-assisted laser desorption/ionisation time-of-flight (MALDI-TOF) mass spectrometry (VITEK MS, bioMérieux). Antimicrobial susceptibility testing was performed by the hospital microbiology laboratory, which is accredited to [41], by disc diffusion on Mueller–Hinton agar with a 0.5 McFarland inoculum according to the EUCAST method, or with VITEK 2 AST cards for selected isolates, and was interpreted against the EUCAST breakpoint tables in force at the time of testing, v14.0 (2024) [42] and v15.0 (2025) [43]. ESBL production in Enterobacterales was confirmed by the double-disc synergy test described in the EUCAST guidelines for the detection of resistance mechanisms [44] (amoxicillin–clavulanate central disc, with cefotaxime, ceftazidime and cefepime discs at 20–30 mm) or by the VITEK 2 ESBL test; AmpC production was not tested. Colistin susceptibility was determined in multidrug-resistant isolates by broth microdilution (UMIC Colistine, Bruker Daltonics, Bremen, Germany) or VITEK 2, and disc diffusion was not used for colistin. Carbapenemase production was investigated in selected carbapenem-resistant isolates by the modified Hodge test, the Carba NP test and the RESIST-5 O.K.N.V.I. immunochromatographic assay (Coris BioConcept, Gembloux, Belgium), which identifies OXA-48-like, KPC, NDM, VIM and IMP enzymes. All testing was performed in the hospital laboratory; carbapenemase results are not recorded in the surveillance registry and are not analysed here.

The XDR designation was assigned not by the laboratory but by the reporting physicians and the infection prevention and control team under the hospital surveillance reporting protocol, on the basis of the laboratory antibiogram: an isolate was designated XDR when it remained susceptible to no more than two of the antimicrobial classes tested for that isolate, polymyxins being counted as a retained class when colistin susceptibility had been demonstrated. This rule adopts the threshold of the ECDC/CDC 2012 interim standard definition of XDR, namely non-susceptibility to at least one agent in all but two or fewer antimicrobial categories [23], but evaluates it against a different denominator. Two aspects of this correspondence require explicit statement. First, the threshold was applied to the pharmacological classes actually tested for each isolate rather than to the fixed, organism-specific category lists tabulated by Magiorakos et al., which comprise 17 antimicrobial categories for *Enterobacteriaceae*, 9 for *Acinetobacter* spp. and 8 for *Pseudomonas aeruginosa* and which that framework requires to be tested in full or nearly in full, with selective reporting avoided [23]; the protocol counts β-lactam classes separately (penicillins, penicillin–inhibitor combinations, cephalosporins, monobactams and carbapenems). The framework introduced those organism-specific categories, each enumerated in a dedicated table, because no standard approach existed for defining resistance in terms of antimicrobial classes [23]; its criteria are nonetheless widely rendered in terms of classes rather than categories in the subsequent literature [5,26], and have also been applied at the level of individual agents [4]. The classification used here follows that class-based convention and is therefore related to, but not interchangeable with, category-based application of the framework. Because a routine susceptibility panel comprises fewer classes than the complete category list, the threshold of two or fewer remaining classes is reached more readily, and the XDR proportions reported here are expected to be higher than those obtained under a strict category-based application. One divergence acts in the opposite direction: a result reported as intermediate was counted as a class retained, following the EUCAST convention that an intermediate result denotes susceptibility at increased exposure, whereas the 2012 framework counts an intermediate result as non-susceptibility. The net effect of the two cannot be determined from the fields retained in the registry. Second, the classification was assigned prospectively during routine surveillance and was not re-derived by the investigators, as neither the composition of the panel nor category-level susceptibility profiles were retained in the registry. The XDR designation is accordingly reported as surveillance-assigned.

The multidrug-resistant (MDR) classification of the same framework, which requires non-susceptibility in three or more antimicrobial categories, could not be derived from the available fields and is not reported.

The framework requires that categories to which a species is intrinsically resistant be removed before the definitions are applied [23]. The hospital reporting protocol applies this step, but because the registry stores assigned phenotypes rather than category-level profiles, it could not be verified retrospectively; among the Gram-negative isolates, three *Stenotrophomonas maltophilia* isolates were recovered, of which one carries a carbapenem-resistance flag although the species is intrinsically non-susceptible to carbapenems. These three isolates represent 1.2% of the 243 Gram-negative isolates and their reclassification would not materially alter any reported proportion.

The registry records a single resistance marker per Gram-negative isolate, assigned hierarchically (XDR > CARBA > ESBL). In the reporting practice of this hospital an XDR designation is given only to carbapenem-resistant isolates, so that the CARBA field identifies carbapenem-resistant isolates that remained susceptible to three or more classes and therefore did not meet the XDR threshold; the two fields are accordingly mutually exclusive, and no Gram-negative isolate carried both (0/243; Table S1). The same hierarchy means that ESBL production is not recorded for carbapenem-resistant isolates, and ESBL proportions are therefore reported among carbapenem-susceptible Enterobacterales. The primary endpoint, carbapenem-resistant Gram-negative infection (CR-GN), was therefore derived as the union of the two fields restricted to Gram-negative isolates, and was further disaggregated into the standard surveillance categories CRE, CRAB and CRPA. At patient level, an outcome was considered present if at least one qualifying isolate was recorded.

Antimicrobial exposure is recorded at the patient level as the agents administered during the hospitalisation in which the HAI occurred, without attribution to a specific infection and without timing relative to culture sampling. For the stewardship analysis, patients who received at least one carbapenem were classified into four mutually exclusive groups on the basis of the isolates recorded for them: those with at least one carbapenem-resistant Gram-negative isolate; those with no carbapenem-resistant Gram-negative isolate but at least one extended-spectrum β-lactamase-producing, carbapenem-susceptible Enterobacterales isolate, for which carbapenem therapy is guideline-concordant; those with at least one Gram-negative isolate but none meeting either condition; and those with no Gram-negative isolate. Carbapenems are not active against carbapenem-resistant isolates, so the first group identifies patients whose recovered organism was not covered by the carbapenem rather than patients with an indication for it. Because the registry does not record the timing of antimicrobial administration relative to culture sampling, the third group identifies patients in whom a narrower alternative was documented, not patients in whom de-escalation demonstrably failed to occur.

### 4.5. Charlson Comorbidity Index

The CCI was computed from the recorded comorbidities using the original Charlson weights [45]. It is summarised as a median with an interquartile range and was entered into the regression models dichotomised at six points, a cut-off applied in other hospitalised cohorts and shown there to be independently associated with mortality [46,47].

### 4.6. Statistical Analysis

Continuous variables were summarised as median [IQR] and compared with the Mann–Whitney U test; categorical variables as n (%) and compared with the Pearson χ^2^ test with Yates’ continuity correction, or Fisher’s exact test when any expected cell count was below five. Inferential comparisons were made between the two Gram-negative strata only (Section 4.1); the third stratum is presented descriptively. *p*-values are descriptive and no adjustment for multiplicity was applied.

Associations were estimated by binary logistic regression and reported as odds ratios with Wald 95% confidence intervals. Eight candidate variables were examined in unadjusted comparisons: age ≥ 65 years, sex, area of residence, intensive care transfer, length of stay > 14 days, Charlson index ≥ 6, carbapenem exposure and receipt of three or more antibiotics. Because the smaller Gram-negative stratum contained 46 patients, a model containing all eight would have provided 5.8 events per variable, below the conventional threshold of ten [48]; the adjusted model was therefore restricted to three exposures—intensive care transfer, carbapenem exposure and receipt of three or more antibiotics—selected on clinical and literature grounds rather than from the observed associations, giving 15.3 events per variable. The mortality model, fitted in the same Gram-negative population, contained six covariates and provided 13.5 events per variable.

Model performance was assessed by the area under the receiver operating characteristic (ROC) curve with DeLong 95% confidence intervals, the Hosmer–Lemeshow goodness-of-fit test with ten groups, and variance inflation factors. Temporal trend in the proportion of carbapenem-resistant isolates was assessed by the Cochran–Armitage test for trend across the eight calendar quarters, by logistic regression with quarter entered as a continuous term, and by a χ^2^ test of homogeneity between quarters; quarterly proportions are reported with exact binomial confidence intervals. Reserve-antibiotic safety context used published EudraVigilance reporting odds ratios [16].

Analyses were performed in R version 4.6.0 [49], using the packages readxl, dplyr and tidyr for data handling, pROC for ROC curve analysis, car for variance inflation factors, DescTools for the Cochran–Armitage trend test, ResourceSelection for the Hosmer–Lemeshow test, and ggplot2 for graphics. Two-sided *p* < 0.05 was considered statistically significant.

During data validation, three isolates carrying an XDR flag on a non-Gram-negative organism were identified as data-entry errors and corrected to negative. As the resistance endpoints are restricted to Gram-negative isolates, the correction did not alter any reported result.

### 4.7. Ethical Approval

The study used exclusively pre-existing, irreversibly anonymized data collected during routine, legally mandated HAI surveillance (Romanian Ministry of Health Order 1101/2016; Law 95/2006 on healthcare reform); no additional diagnostic or therapeutic procedure was performed and no identifiable personal data were processed. The processing of fully anonymized surveillance data for scientific research is compatible with Article 9(2)(j) of the EU General Data Protection Regulation (Regulation 2016/679) and Article 7 of Romanian Law 190/2018, and, for irreversibly anonymized data, falls outside the material scope of data-protection consent requirements per Romanian National Supervisory Authority (ANSPDCP) guidance. Ethics committee approval and the waiver of individual informed consent are reported in the Institutional Review Board Statement and the Informed Consent Statement.

## 5. Conclusions

At this centre, carbapenem resistance in Gram-negative healthcare-associated infection is endemic: it persisted at a high level throughout the 24 months and was not concentrated in any patient group that routine data can identify. The excess relative to European surveillance is confined to the Enterobacterales, the two non-fermenting genera falling within the range reported by countries of comparable burden. Because more than nine in ten resistant isolates retained no more than two active antimicrobial classes, and because no routinely recorded patient characteristic separated resistant from susceptible infection to a degree that would alter empirical treatment, risk-based targeting appears to offer little in this setting; the available lever is prospective audit of carbapenem prescribing, including susceptibility-based de-escalation, rather than patient-level prediction. Resistance status was not independently associated with in-hospital mortality after adjustment for intensive care exposure and comorbidity burden, although the susceptible comparison group was too small to exclude a moderate effect. These conclusions describe a tertiary referral centre for infectious diseases and are conditioned on an extensively drug-resistant designation assigned prospectively under the hospital surveillance protocol against the antimicrobial classes tested for each isolate rather than against a fixed category list, and on antibiotic exposure recorded without timing relative to culture sampling. Retaining the agents tested and their results, together with carbapenemase type, in the surveillance record would allow the same routine data to support classification comparable with European surveillance and prospective audit of the de-escalation opportunity quantified here, and would establish whether the absence of an association between resistance and mortality persists once resistance mechanism is taken into account.

## Figures and Tables

**Table 1:** Cohort characteristics by stratum.

Variable	Overall (*n* = 315)	A: CR-GN (*n* = 118)	B: GN Non-CR (*n* = 46)	C: No GN (*n* = 151)	*p* (A vs. B) ^1^
Age, years, median [IQR]	69 [58–77]	69 [62–76]	68.5 [57.2–77.8]	69 [56–79]	0.799
Age ≥ 65 years, n (%)	200 (63.5)	79 (66.9)	27 (58.7)	94 (62.3)	0.417
Male sex, n (%)	187 (59.4)	76 (64.4)	27 (58.7)	84 (55.6)	0.617
Rural residence, n (%)	159 (50.5)	67 (56.8)	23 (50.0)	69 (45.7)	0.542
ICU transfer, n (%)	93 (29.5)	58 (49.2)	14 (30.4)	21 (13.9)	0.046
ICU stay, days, median [IQR]	0 [0–4]	0 [0–15.8]	0 [0–4]	0 [0–0]	0.024
Total hospital stay, days, median [IQR]	18 [11–25.5]	21 [13–31]	16 [12.2–25]	15 [11–22]	0.133
Days to first HAI, median [IQR]	8 [5–12.5]	9.5 [6–15]	8 [5–12.8]	7 [3–10]	0.150
≥2 HAI episodes, n (%)	64 (20.3)	40 (33.9)	13 (28.3)	11 (7.3)	0.612
Charlson index, median [IQR]	5 [3–7]	5 [4–7]	5 [2.5–7]	5 [3–7]	0.556
Charlson index ≥ 6, n (%)	132 (41.9)	56 (47.5)	18 (39.1)	58 (38.4)	0.431
Number of antibiotics, median [IQR]	2 [1–3]	3 [2–4]	2 [2–3]	1 [1–2]	0.084
≥3 antibiotics, n (%)	93 (29.5)	60 (50.8)	16 (34.8)	17 (11.3)	0.093
Carbapenem exposure, n (%)	92 (29.2)	54 (45.8)	17 (37.0)	21 (13.9)	0.397
In-hospital death, n (%)	122 (38.7)	62 (52.5)	19 (41.3)	41 (27.2)	0.263

^1^ Mann–Whitney U test for continuous variables and Pearson χ^2^ test with Yates’ correction, or Fisher’s exact test where an expected cell count was below five, for categorical variables. *p*-values compare strata A and B only, the sole contrast that isolates resistance rather than organism type; they are descriptive and no adjustment for multiplicity was applied. Stratum C is presented as context and is not assigned a *p*-value. CR, carbapenem-resistant; CR-GN, carbapenem-resistant Gram-negative; GN, Gram-negative; HAI, healthcare-associated infection; ICU, intensive care unit; IQR, interquartile range.

**Table 2:** Carbapenem resistance and extensive drug resistance among Gram-negative isolates, by species.

Species	*n*	CR, *n*	CR, %	XDR, *n*	XDR, %	XDR as % of CR
*K. pneumoniae*	96	87	90.6	84	87.5	96.6
*A. baumannii*	53	44	83.0	42	79.2	95.5
*P. aeruginosa*	51	17	33.3	11	21.6	64.7
*E. coli*	15	0	0.0	0	0.0	—
*P. mirabilis*	6	3	50.0	3	50.0	100.0
*Proteus* spp.	5	1	20.0	1	20.0	100.0
*S. marcescens*	4	2	50.0	2	50.0	100.0
Other Gram-negative species (10 species)	13	8	61.5	7	53.8	87.5
All Gram-negative isolates	243	162	66.7	150	61.7	92.6

Species with at least four isolates are listed individually; the remaining ten species are pooled. XDR isolates are a subset of carbapenem-resistant isolates: in the reporting practice of this hospital, an XDR designation implies carbapenem resistance (Section 4.4), and no isolate carried both the CARBA and the XDR registry fields. Percentages in the CR and XDR columns are of isolates of the species; the final column expresses XDR isolates as a percentage of carbapenem-resistant isolates of that species. CR, carbapenem-resistant; XDR, extensively drug-resistant.

**Table 3:** Carbapenem resistance among Gram-negative isolates by infection site, specimen and species.

Infection Site (Episodes)	Specimens (Gram-Negative Isolates)	*K. pneumoniae*	*A. baumannii*	*P. aeruginosa*	*E. coli*	Other	CR, *n*/*N* (%)
Lower respiratory tract (109)	Sputum 50; tracheobronchial aspirate 46; bronchial aspirate 31; pleural fluid 2	27/35	30/37	9/34	0/6	8/17	74/129 (57.4)
Urinary tract (47)	Urine 47; urethral secretion 1; urinary catheter tip 1	29/30	4/4	3/5	0/5	4/5	40/49 (81.6)
Skin and soft tissue (19)	Pressure-ulcer secretion 21; purulent secretion 14	15/15	3/4	2/8	0/3	1/5	21/35 (60.0)
Sepsis (19)	Blood culture 18; pressure-ulcer secretion 2	14/14	4/5	—	0/1	—	18/20 (90.0)
Venous catheter (6)	Catheter tip 8	2/2	3/3	2/2	—	1/1	8/8 (100.0)
Other (2)	Conjunctival secretion 1; vaginal secretion 1	—	—	1/2	—	—	1/2 (50.0)
All sites (202)	243	87/96	44/53	17/51	0/15	14/28	162/243 (66.7)

Cells show carbapenem-resistant isolates/all isolates of the species at that site. Infection site and specimen are recorded per episode; in the 33 episodes that yielded more than one Gram-negative isolate, all isolates are attributed to the specimen recorded for the episode. In one episode of sepsis that originated from an infected pressure ulcer, the specimen recorded was the pressure-ulcer secretion, which yielded two Gram-negative isolates. Episode counts refer to episodes yielding at least one Gram-negative isolate; the 107 gastrointestinal episodes yielded none. Other: 28 isolates of 13 further species. CR, carbapenem-resistant.

**Table 4:** Exploratory comparison of patients with carbapenem-resistant and carbapenem-susceptible Gram-negative infection (n = 164).

Variable	A: CR-GN (*n* = 118), *n* (%)	B: GN Non-CR (*n* = 46), *n* (%)	Crude OR (95% CI)	*p*	Adjusted OR (95% CI) ^1^	*p*
Age ≥ 65 years	79 (66.9)	27 (58.7)	1.43 (0.71–2.87)	0.417	—	—
Male sex	76 (64.4)	27 (58.7)	1.27 (0.63–2.56)	0.617	—	—
Rural residence	67 (56.8)	23 (50.0)	1.31 (0.66–2.60)	0.542	—	—
ICU transfer	58 (49.2)	14 (30.4)	2.21 (1.07–4.56)	0.046	1.86 (0.83–4.18)	0.132
Length of stay > 14 days	79 (66.9)	27 (58.7)	1.43 (0.71–2.87)	0.417	—	—
Charlson index ≥ 6	56 (47.5)	18 (39.1)	1.41 (0.70–2.81)	0.431	—	—
Carbapenem exposure	54 (45.8)	17 (37.0)	1.44 (0.71–2.90)	0.397	0.99 (0.45–2.19)	0.983
≥3 antibiotics	60 (50.8)	16 (34.8)	1.94 (0.96–3.93)	0.093	1.51 (0.66–3.42)	0.328

^1^ Adjusted odds ratios are from a single logistic model containing intensive care transfer, carbapenem exposure, and receipt of three or more antibiotics (15.3 events per variable; area under the curve 0.618, 95% CI 0.529–0.707; maximum variance inflation factor 1.33). Variables marked—were not entered because with 46 patients in the smaller stratum a model containing all eight candidate variables would have provided 5.8 events per variable. *p*-values are descriptive and unadjusted for multiplicity. CI, confidence interval; CR-GN, carbapenem-resistant Gram-negative; GN, Gram-negative; ICU, intensive care unit; OR, odds ratio.

**Table 5:** Antimicrobial stewardship indicators.

Indicator	*n* (%)
Patients receiving ≥ 1 carbapenem	92/315 (29.2)
with a carbapenem-resistant Gram-negative isolate (not covered by carbapenems)	54/92 (58.7)
of whom also receiving colistin, tigecycline or cefiderocol	34/54 (63.0)
with an ESBL-producing, carbapenem-susceptible isolate	5/92 (5.4)
with a Gram-negative isolate, neither carbapenem-resistant nor ESBL-producing	12/92 (13.0)
with no Gram-negative isolate	21/92 (22.8)
Patients receiving ≥ 1 Reserve antibiotic (WHO AWaRe)	102/315 (32.4)
Patients receiving ≥ 3 antibiotics	93/315 (29.5)
Patients with Charlson index ≥ 6 exposed to a Reserve antibiotic	49/315 (15.6)

Carbapenem recipients are classified into four mutually exclusive groups that sum to 92. Carbapenem therapy is guideline-concordant for ESBL-producing Enterobacterales, so those patients are listed separately from patients whose Gram-negative isolates were neither carbapenem-resistant nor ESBL-producing. Carbapenems are not active against carbapenem-resistant isolates, so the first group identifies patients whose recovered organism was not covered by the carbapenem, not patients with an indication for it. Because the registry does not record the timing of antibiotic administration relative to culture sampling, the group with a Gram-negative isolate that was neither carbapenem-resistant nor ESBL-producing identifies patients in whom de-escalation was possible in principle, not patients in whom it failed to occur. Reserve antibiotics under the 2023 World Health Organisation (WHO) Access, Watch, Reserve (AWaRe) classification available in this cohort were colistin, cefiderocol, linezolid, tigecycline, fosfomycin and aztreonam. ESBL, extended-spectrum β-lactamase.

**Table 6:** Cohort exposure to last-resort antibiotics and corresponding published EudraVigilance signals (Vintila et al. [16]).

Antibiotic (AWaRe)	Cohort Exposure, *n* (%)	EU ICSRs	% DR/% DI Reports	% Fatal (DR)	Key ROR (95% CI) ^1^
Vancomycin (Watch)	165 (52.4)	—	—	—	comparator drug
Meropenem/carbapenem (Watch)	92 (29.2)	8864	3.56/9.45	20	3.24 (2.91–3.61) DI vs. MOX
Colistin (Reserve)	73 (23.2)	986	8.42/10.14	24	16.91 (12.57–22.74) DR vs. MOX
Linezolid (Reserve)	36 (11.4)	13,381	2.38/4.15	6	1.34 (1.20–1.51) DI vs. MOX
Tigecycline (Reserve)	29 (9.2)	—	—	—	comparator drug
Cefiderocol (Reserve)	5 (1.6)	—	—	—	not analysed [16]

^1^ Reporting odds ratio (ROR) values and report counts are reproduced from Vintila et al. [16] (EudraVigilance, data to 31 December 2022) and are external to this cohort. DR, drug resistance; DI, drug ineffectiveness; MOX, moxifloxacin comparator. Cohort exposure figures are from the present study. AWaRe, WHO Access, Watch, Reserve classification; ICSR, Individual Case Safety Report.

## Data Availability

Restrictions apply to the availability of these data. The data were obtained from the healthcare-associated infection surveillance registry of the “Dr. Victor Babeș” Clinical Hospital of Infectious Diseases and Pneumophthisiology, Timișoara, Romania, and are available from the authors only with the permission of that institution and its Ethics Committee. A further restriction applies independently of ownership: although direct identifiers were removed before analysis, the records retain a combination of admission period, ward, comorbidity and outcome variables from which re-identification at record level cannot be excluded in a single-centre cohort of 315 patients.

## Supplementary Materials

The following supporting information can be downloaded at: https://www.mdpi.com/article/10.3390/antibiotics15101003/s1, Table S1: Contingency of the CARBA and XDR registry fields among the 243 Gram-negative isolates.

## Abbreviations

The following abbreviations are used in this manuscript:

AWaRe	World Health Organisation Access, Watch, Reserve classification of antibiotics
CARBA	Carbapenem-resistant (surveillance registry field)
CCI	Charlson Comorbidity Index
CDC	Centres for Disease Control and Prevention
CI	Confidence interval
CR	Carbapenem-resistant
CR-GN	Carbapenem-resistant Gram-negative
CRAB	Carbapenem-resistant *Acinetobacter baumannii*
CRE	Carbapenem-resistant Enterobacterales
CRPA	Carbapenem-resistant *Pseudomonas aeruginosa*
DI	Drug ineffectiveness (adverse-reaction term)
DR	Drug resistance (adverse-reaction term)
EARS-Net	European Antimicrobial Resistance Surveillance Network
ECDC	European Centre for Disease Prevention and Control
ESBL	Extended-spectrum β-lactamase
EU/EEA	European Union/European Economic Area
EUCAST	European Committee on Antimicrobial Susceptibility Testing
GN	Gram-negative
HAI	Healthcare-associated infection
HAI-Net	Healthcare-Associated Infections Surveillance Network
ICSR	Individual Case Safety Report
ICU	Intensive care unit
IMP	Imipenemase metallo-β-lactamase
IQR	Interquartile range
KPC	*Klebsiella pneumoniae* carbapenemase
MALDI-TOF	Matrix-assisted laser desorption/ionisation time-of-flight
MDR	Multidrug-resistant
MOX	Moxifloxacin (pharmacovigilance comparator)
NDM	New Delhi metallo-β-lactamase
NHSN	National Healthcare Safety Network
ONPG	ortho-Nitrophenyl-β-D-galactopyranoside
OR	Odds ratio
OXA-48	Oxacillinase-48 carbapenemase
ROC	Receiver operating characteristic
ROR	Reporting odds ratio
SOFA	Sequential Organ Failure Assessment
VIM	Verona integron-encoded metallo-β-lactamase
WHO	World Health Organisation
XDR	Extensively drug-resistant
