# Supplementary material for: Carbapenem-Resistant and Extensively Drug-Resistant Gram-Negative Healthcare-Associated Infections in a Romanian Tertiary Infectious Diseases Hospital: Phenotypic Landscape, Stewardship Gaps and Outcomes (2024–2025)
Source: Antibiotics (Basel). 2026 Oct 10;15(10):1003. doi: 10.3390/antibiotics15101003 (PMC13700804; doi:10.3390/antibiotics15101003)
Supplement: Supplementary file 1 [file antibiotics-15-01003-s001.zip › antibiotics-4552629-supplementary.pdf]

Supplementary Materials

Carbapenem-Resistant and Extensively Drug-Resistant Gram-Negative Healthcare-Associated Infections in a Romanian Tertiary Infectious Diseases Hospital: Phenotypic Landscape, Stewardship Gaps and Outcomes (2024–2025)

Table S1. Contingency of the CARBA and XDR registry fields among the 243 Gram-negative isolates.

	XDR = 0	XDR = 1
CARBA = 0	81	150
CARBA = 1	12	0

The registry records a single resistance marker per Gram-negative isolate, assigned hierarchically (XDR > CARBA > ESBL). The XDR designation is assigned by the reporting physicians and the infection prevention and control team under the hospital surveillance protocol; in the reporting practice of this hospital an XDR designation is given only to carbapenem-resistant isolates, whereas the CARBA field, determined by the laboratory, denotes carbapenem-resistant isolates that remained susceptible to three or more classes; the two fields are therefore mutually exclusive. No isolate carried both flags. The primary endpoint, carbapenem-resistant Gram-negative infection, is the union of the two fields restricted to Gram-negative isolates (162 of 243).
